# Coinfection of Typhoid Fever With Tuberculosis: A Challenge to Surgical Management

**DOI:** 10.7759/cureus.8540

**Published:** 2020-06-09

**Authors:** Anusha Dudaka, Sudharsanan Sundaramurthi, Chellappa Vijayakumar, TP Elamurugan, Sadasivan Jagdish

**Affiliations:** 1 Surgery, Jawaharlal Institute of Postgraduate Medical Education and Research, Puducherry, IND; 2 Surgery, Pondicherry Institute of Medical Sciences, Puducherry, IND

**Keywords:** small bowel, anastomosis, ileostomy, non-traumatic perforation, resuscitation, lag period

## Abstract

Ileal perforation is one of the most dreaded complications of abdominal tuberculosis. It is more common in immunodeficient patients, where ulcerative type of intestinal tuberculosis predominates. Various factors play role in the outcome of these patients, such as age and comorbid illness, though the lag period (advent of symptoms to time of admission to hospital) correlated directly to the mortality in these patients. Herein we present a 28-year-old male who had a coinfection of typhoid fever along with intestinal tuberculosis. The patient presented with abdominal pain and fever for one-week duration. On examination, he had diffuse tenderness of his abdomen with guarding. X-ray revealed free air under diaphragm. The patient underwent limited resection of terminal ileum and cecum with end ileostomy for ileal perforation. The patient’s serum Widal test was positive and blood culture grew *Salmonella *Typhi, and the patient was started on intravenous (IV) antibiotics based on culture and sensitivity. The patient’s general condition worsened after two weeks with bile leak from the surgical site. The patient succumbed to severe sepsis. Postoperative histopathology of the resected ileo-cecal segment showed features of ileo-cecal tuberculosis. As typhoid is a common cause of ileal perforation in the developing countries, the co-existence of typhoid fever in this patient lead to the delay in the diagnosis and appropriate management of tubercular ileal perforation. Knowledge about various causes of typhoid perforation is essential for treating surgeons.

## Introduction

Ileal perforation is one of the most dreaded complications of abdominal tuberculosis. It is more common in immunodeficient patients, where the ulcerative type of intestinal tuberculosis predominates. Patients with tubercular ileal perforations have high mortality of up to 30% in various studies [1]. Various factors play a role in the outcome of these patients, such as age and comorbid illness, though the lag period (the advent of symptoms to time of admission to hospital) correlated directly to the mortality in these patients [2]. Late presentation results in extensive pathological changes in the ileum and cecum, leading to increased morbidity and mortality. Herein, we present a 28-year-old male who had a coinfection of typhoid fever along with intestinal tuberculosis.

This work has been presented as an abstract (Case report: Anusha D, Sudharsanan S, Elamurugan TP, Jagdish S. Typhoid Fever Masquerading Tubercular Ileal Perforation - A Rare Case Report. XXVII National Conference of Indian Association of Surgical Gastroenterology; 2017/06/12).

## Case presentation

A 28-year-old male presented to the emergency room with complaints of diffuse abdominal pain, low-grade fever, and vomiting of seven days' duration. He had no history of non-steroidal anti-inflammatory drug abuse. He had no known comorbidities. On examination, the patient was emaciated, and dehydrated with tachycardia and tachypnea. His abdomen was distended with diffuse tenderness and guarding.

**Figure 1:**
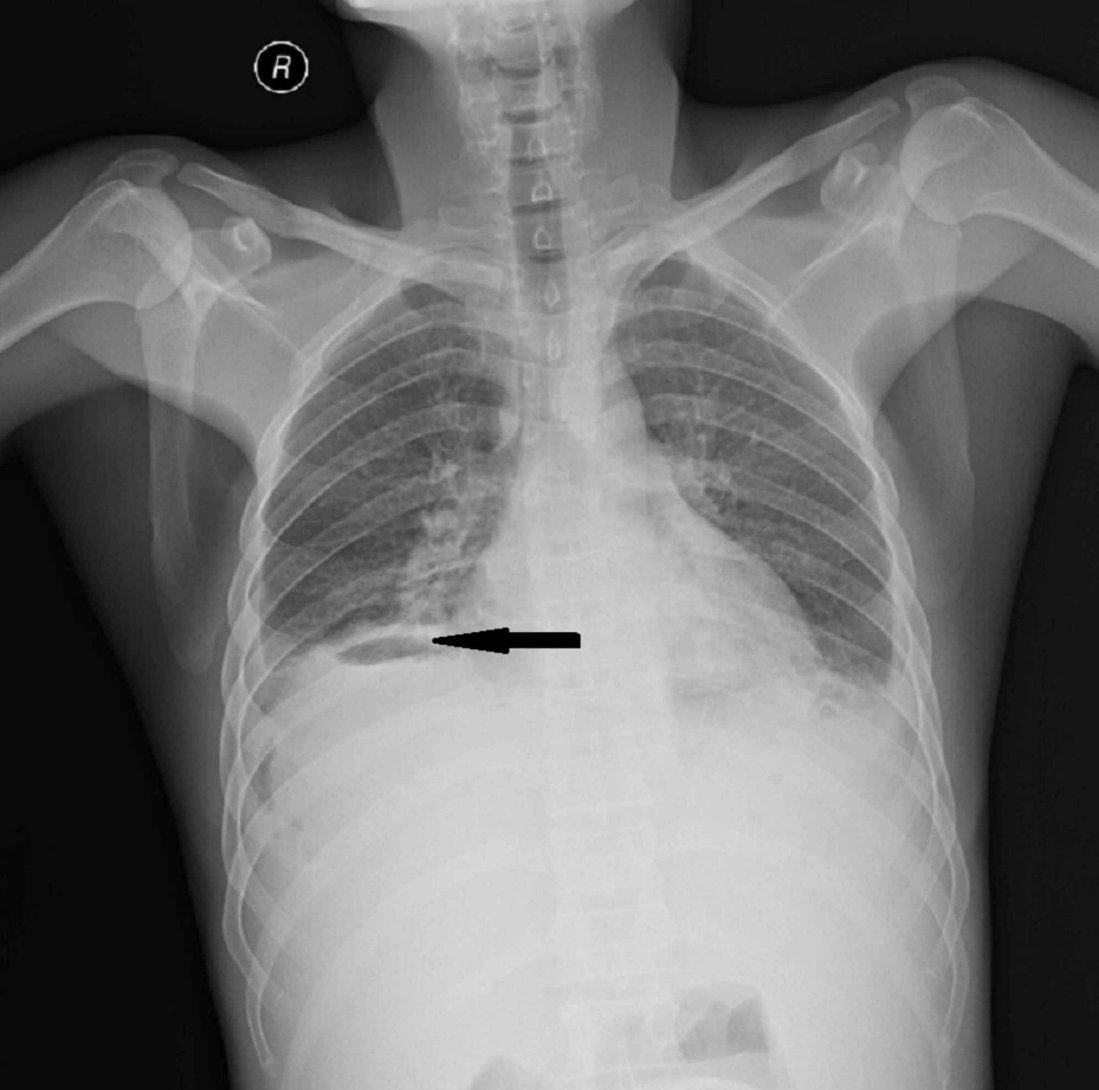
Erect chest X-ray showing free air under the diaphragm (arrow), a feature of bowel perforation.

Chest X-ray showed air under the diaphragm, and he was taken up for exploratory laparotomy (Figure 1).

Intra-operatively, approximately two liters of biliopurulent contamination was noted. A 0.5-cm perforation was identified in the terminal ileum, about 20 cm from the ileo-cecal junction. Cecum and terminal ileum were unhealthy, so a limited resection of the cecum along with terminal ileum and a double-barrel ileostomy was done. No other perforations noted in the remaining bowel.

Postoperatively, he received intravenous (IV) cefoperazone-sulbactam, metronidazole, and gentamicin. His aerobic blood culture grew *Salmonella *Typhi*, *which was sensitive to ampicillin; his serum Widal test showed positive titers. With the diagnosis of typhoid fever causing ileal perforation, he was administered a 14-day course of ampicillin.

Postoperatively, the ileostomy was functioning well and he was started on oral feeds. On postoperative day 12, he had biliopurulent discharge from the surgical site. Ultrasound abdomen showed a small interbowel collection, which was communicating through a defect in the abdominal wall to the incision site. Abdominal sutures were removed, which revealed complete wound dehiscence with continuous discharge from the peritoneal cavity. In view of high output from the stoma and poor nutritional status (albumin 1.9 mg/dl, weight 30 kg), he was started on total parenteral nutrition. His general condition progressively worsened, and he expired after one month of hospital stay.

**Figure 2:**
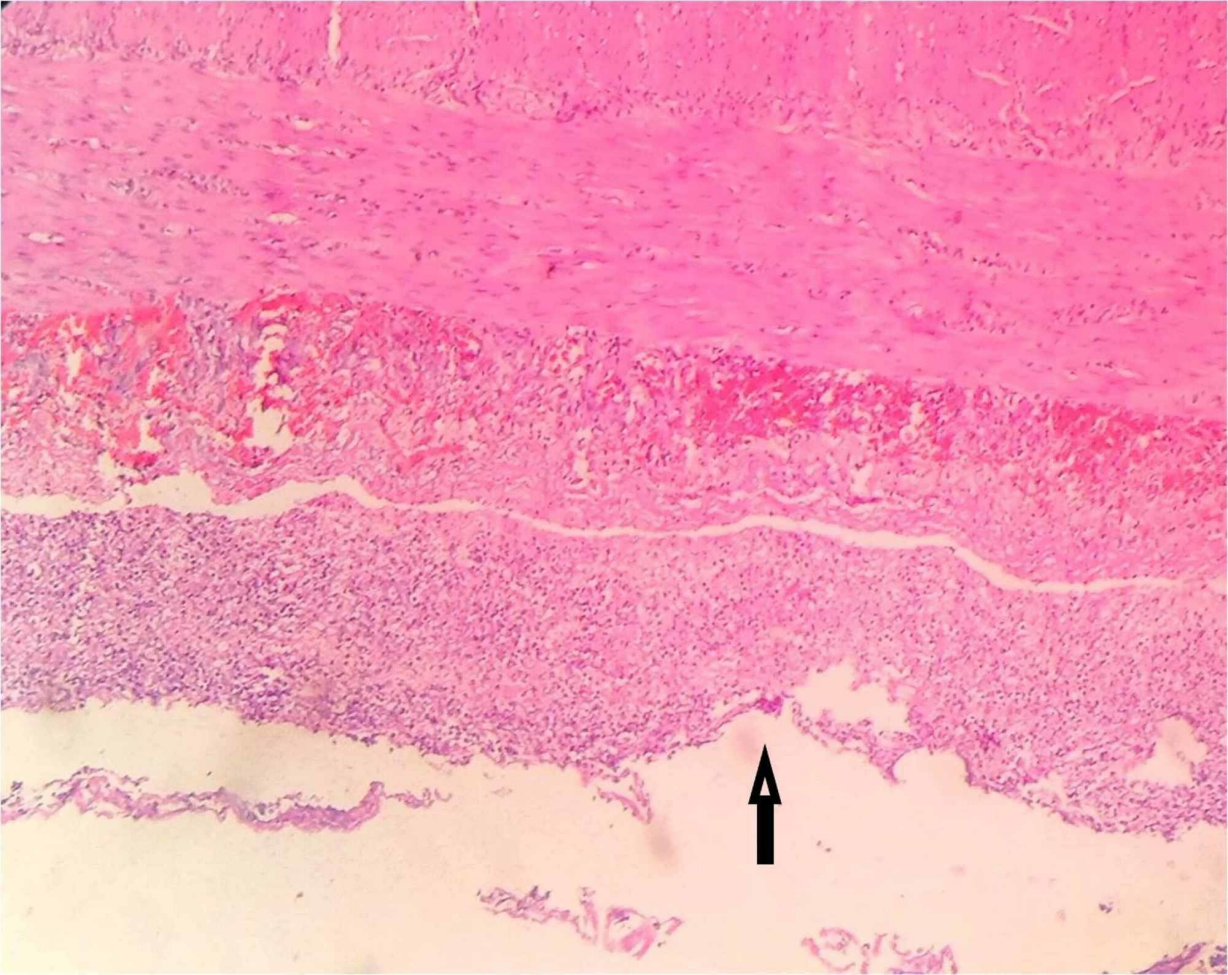
Microscopic examination of small bowel showing ulcer in the mucosa (arrow) with areas of necrosis, transmural inflammation with acute inflammatory cells and serositis. X100.

**Figure 3:**
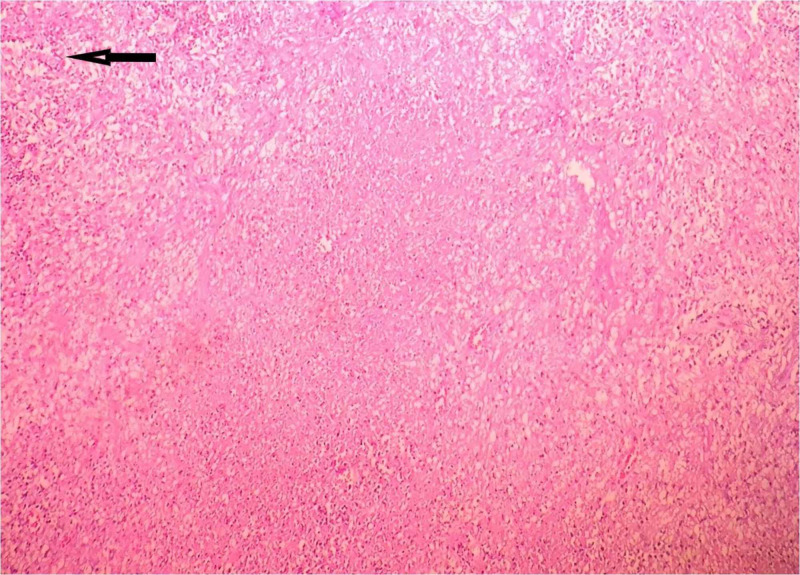
Microscopic examination of mesenteric lymph node showing collection of foamy macrophages, epithelioid cells, caseous necrosis, and positive staining for acid-fast bacilli (arrow). X100.

Histopathological examination of the resected specimen showed features of intestinal tuberculosis with perforation. Microscopic examination of small bowel showed ulceration in the mucosa with areas of necrosis, transmural inflammation with acute inflammatory cells, and serositis. Some lymph nodes showed a collection of foamy macrophages, epithelioid cells along with caseous necrosis, and staining for acid-fast bacilli was positive (Figures 2, 3).

## Discussion

Ileal perforation is one of the commonest abdominal emergencies in developing countries. Most ileal perforations are of traumatic origin. Non-traumatic ileal perforation is usually caused by malignancies, Crohn’s disease, hernias, radiotherapy, bands, and other mechanical causes in the Western countries. Infectious conditions, such as typhoid and tuberculosis, are the common causes of non-traumatic ileal perforation in developing countries [3]. In spite of advances in diagnostic and therapeutic modalities, this condition carries high morbidity as well as mortality. 

Ileo-cecal region is a common extrapulmonary site of affection for tuberculosis. The organism reaches the terminal ileum either by the ingestion of infected sputum and localizing in the Payer’s patches or directly by hematogenous route. The pathological types include hyperplastic, ulcerative, and mixed or the stricturous type [4]. The hyperplastic type leads to mass or stricture formation and usually presents with intestinal obstruction. The ulcerative type complicates by tubercular perforation, especially in immunocompromised patients. The perforation is solitary in 90% of the cases [1]. In our patient, there was a single perforation involving the terminal ileum. 

The clinical features of tubercular perforation are non-specific. The most common symptom of ileo-cecal tuberculosis is intermittent abdominal pain, which is usually present over a few months. Sudden worsening of abdominal pain associated with abdominal distension usually suggests bowel perforation. The patients can have associated vomiting, constipation, and fever [5]. Passage of melenic stools can be seen in a few patients. The patient is emaciated in most cases and present with signs of toxemia with tachycardia and hypotension. On abdominal examination, diffuse tenderness, guarding, and rigidity are usually present as seen in our patient. 

Chest and abdomen X-ray erect view can show features of pneumoperitoneum seen as free air under the diaphragm. Cavitations in the lung can be seen in patients with primary pulmonary tuberculosis. Ultrasound evaluation of the abdomen can reveal free fluid with particulate matter, bowel dilatation, and enlarged mesenteric lymph nodes [6]. Contrast-enhanced computed tomography (CECT) is not routinely done in patients who present with features of bowel perforation as it leads to delay in the definitive management of the patient. In our patient, the chest X-ray revealed pneumoperitoneum, and hence a CT scan was not done. 

The initial step in management is adequate resuscitation with IV fluids and antibiotics. Nasogastric decompression of the bowel should be done. Urine output and vitals should be monitored at regular intervals. After initial resuscitation, the patient is taken up for explorative laparotomy. Pus from the peritoneal cavity is sent for aerobic culture and antibiotic sensitivity. Thorough lavage of abdomino-pelvic cavity is done to get rid of all the contaminants. The presence of tubercles on the bowel and on the mesentery can suggest a tubercular etiology. The various surgical procedures described are simple repair of perforation after debriding the edges of the perforated site, resection of the perforated segment and primary anastomosis, resection of the segment, and creating a double-barrel stoma [7]. 

The simple closure technique has a high rate of leak and fistula formation and should be avoided in tubercular perforation [7]. In patients with strictures associated with the perforation, the strictured segment is always resected. A primary anastomosis of resected ends is done in patients who are vitally stable, with minimal peritoneal contamination, and having bowel without significant edema. Patients who undergo primary anastomosis can develop re-leak or new perforations due to presence of areas of impending perforations in the small bowel. The mucosal involvement of the disease cannot be assessed properly during initial surgery, and hence surgeons should be cautious while deciding on performing a primary anastomosis. Resection with ileostomy has the best success rate among the various techniques described for tubercular ileal perforation. Anti-tubercular therapy should be initiated after surgical treatment for all confirmed cases of tubercular ileal perforation [8]. 

Surgical site infections are seen in more than 50% of the patients undergoing surgical treatment. Other postoperative complications include wound dehiscence (27%) and intra-abdominal collections (10%). Primary repair of perforation and anastomotic repair have a high incidence of leak from the repair site leading to enterocutaneous fistula formation in up to 10% of cases [9]. 

There were no obvious signs of tuberculosis like tubercles or strictures intra-operatively in our patient. Hence, the patient was worked up for typhoid as well as for tuberculosis. As the blood cultures and Widal test were positive for *Salmonella *Typhi, the patient was managed as a case of typhoid ileal perforation with IV antibiotics. The histological confirmation of tuberculosis was available only after three weeks of surgery that led to the delay in the initiation of anti-tubercular therapy. 

The most important factor that determines the prognosis of these patients is the 'lag period' from the onset of symptoms to admission in to the hospital and subsequent surgical treatment. High mortality and morbidity was seen in patients where the lag period was more than 36 hours. Adequate resuscitation is a must before definitive surgical procedure. Increased mortality was seen in patients who underwent surgery without proper resuscitative measures. The amount of purulent contamination, the length of affected segment, the number of perforations, comorbid conditions, and nutritional and immunological status of the patient are other factors that determine the prognosis of patients with ileal perforation. 

## Conclusions

As typhoid is a common cause of ileal perforation in the developing countries, the co-existence of typhoid fever in this patient led to the delay in the diagnosis and appropriate management of tubercular ileal perforation. Knowledge about various causes of typhoid perforation and complete work-up of these patients can produce an improved clinical outcome in patients presenting with ileal perforation. 

## References

[1] Dan D, Islam S, Naraynsingh V (2015). Free perforation of ileal tubercular ulcer: a case report and literature review. Clin Microbiol.

[2] Ugochukwu AI, Amu OC, Nzegwu MA (2013). Ileal perforation due to typhoid fever-review of operative management and outcome in an urban centre in Nigeria. Int J Surg.

[3] Babu GK, Lokesh K (2016). A clinical study on ileal perforation. J Evid Based Med Healthc.

[4] Pattanayak S, Behuria S (2015). Is abdominal tuberculosis a surgical problem?. Ann R Coll Surg Engl.

[5] Poornima R, Venkatesh KL, Goutham MV, Hassan N (2017). Clinicopathological study of Ileal perforation: study in tertiary center. Int Surg J.

[6] Chalya PL, Mabula JB, Koy Koy (2012). Typhoid intestinal perforations at a University teaching hospital in Northwestern Tanzania: a surgical experience of 104 cases in a resource-limited setting. World J Emerg Surg.

[7] Wani RA, Parray FQ, Bhat NA, Wani MA, Bhat TH, Farzana F (2006). Nontraumatic terminal ileal perforation. World J Emerg Surg.

[8] Gupta S, Jayant M, Kaushik R (2013). Free tubercular perforation of the ileum. World J Emerg Med.

[9] Singh G, Dogra BB, Jindal N, Rejintal S (2014). Non-traumatic ileal perforation: a retrospective study. J Family Med Prim Care.

